# Case Report: Subcutaneous ofatumumab for patients with immunosuppressant-dependent or ineffective primary membranous nephropathy

**DOI:** 10.3389/fimmu.2025.1610530

**Published:** 2025-06-30

**Authors:** Jie Chen, De-tian Li, Xin Feng, Bei-Ru Zhang

**Affiliations:** ^1^ Department of Nephrology, Shengjing Hospital of China Medical University, Shenyang, Liaoning, China; ^2^ Department of Nephrology, Liaoning Electric Power Central Hospital, Shenyang, Liaoning, China

**Keywords:** ofatumumab, membrane nephropathy, PLA2R antibody, subcutaenous administration, CD20 monoclonal antibody

## Abstract

CD20 monoclonal antibodies (mAbs), particularly rituximab, have become a preferred treatment for many patients with phospholipase A2 receptor (PLA2R)-related membranous nephropathy (MN). However, some patients either fail to respond to rituximab or experience adverse reactions, indicating that newer-generation CD20 mAbs may offer a more effective alternative. Recently, subcutaneous ofatumumab has been utilized in the treatment of relapsing multiple sclerosis (RMS). This study presents two patients of primary membranous nephropathy (PMN) patients who were treated with subcutaneous ofatumumab. One patient was unresponsive to immunosuppressive therapies, while the other experienced recurrence after drug withdrawal. After ofatumumab therapy, Case 1 achieved PLA2R antibody negativity, and Case 2 showed improvements in renal function and hypoproteinemia. Both cases experienced a reduction in proteinuria. No adverse reactions were reported during the observation period. In conclusion, this study highlights the efficacy and safety of subcutaneous ofatumumab in treating PMN, particularly in patients who have failed or relapsed after conventional therapies.

## Introduction

Membranous nephropathy (MN) is a kidney disease characterized by the deposition of immune complexes on the glomerular basement membrane, resulting in thickening of the capillary walls (1). Its annual incidence rate is about one case per 100,000 individuals, making it one of the most common causes of nephrotic syndrome (NS) globally (2). MN can be classified into primary membranous nephropathy (PMN) and secondary membranous nephropathy (SMN). Approximately 70–80% of PMN cases are associated with anti-phospholipase A2 receptor (PLA2R) antibodies, which are not only useful for diagnosis but also serve as markers for disease severity and treatment response (3, 4). Although spontaneous remissions characterize PMN, approximately 50% of patients remain in NS, and 30% may progress to end-stage renal disease (ESRD) over the course of 10 years (5). Therefore, identifying targeted therapies remains a critical area of ongoing research.

Historically, the first-line treatment for adult *P*MN consisted of alternating steroids and alkylating agents for six months, with cyclosporine or tacrolimus as alternatives (6). However, these therapies are associated with significant adverse effects, including infections, myelosuppression, nephrotoxicity and hyperglycemia, leading to the exploration of B-cell targeted therapies (7). Rituximab, an anti-CD20 monoclonal antibody, has been widely used to treat *P*MN, as it effectively reduces proteinuria and induces remission (8). The KDIGO 2021 clinical practice guideline recommends rituximab as the first-line treatment for moderate or high-risk PMN patients (9). However, 20–40% of patients do not respond to rituximab, and some who initially respond experience relapses, requiring additional treatment (10, 11). Consequently, more studies have begun to explore novel anti-CD20 monoclonal antibodies.

Ofatumumab is a fully human monoclonal IgG1 antibody approved for the treatment of chronic lymphocytic leukemia (CLL) and relapsing multiple sclerosis (RMS). Nonclinical data suggest that ofatumumab may have greater therapeutic potential than rituximab, with lower doses of ofatumumab achieving similar therapeutic effects (12). It is important to note that the dose and route of ofatumumab administration have varied. Previously, ofatumumab (Arzerra) was a bottled liquid solution available in two concentrations: 100mg and 1000mg, typically administered intravenously in doses ranging from 300 to 2000mg over a 28-day period (13). In recent years, subcutaneous ofatumumab (Kesimpta, 20mg) has been approved for marketing in the treatment of RMS (14). The recommended protocol is 20 mg subcutaneously once a week for the first 3 weeks, followed by a maintenance dose of 20 mg monthly thereafter. It is the first B-cell-targeting therapy designed for self-administration at home, following initial training by a healthcare professional. This study presents two *P*MN patients who received subcutaneous ofatumumab (Kesimpta) for the first time, providing clinical evidence for new indications of the drug.

## Case presentation

### Case 1

A 45-year-old man was diagnosed stage 2 *P*MN by renal biopsy five years earlier (October 2019). The main reason for his visit was the incidental finding of proteinuria during a routine physical examination. He had no past history of kidney disease or autoimmune disease, nor any significant medical history such as hypertension or diabetes. The initial urine protein was 5.15 g/day, plasma albumin was 29.8 g/L, and anti-PLA2R antibody was elevated (>100 RU/ml). The patient was started on a regimen of high-dose corticosteroids combined with intravenous cyclophosphamide. Additional medications included losartan, which was later discontinued due to hypotension intolerance (from 110/70mmHg to 95/60mmHg). Due to the requirement for repeated hospitalizations for intravenous cyclophosphamide administration, it was switched to oral cyclosporine A. This regimen was continued for approximately 2 years, plasma albumin was almost normal(38–40 g/L), but anti-PLA2R antibody remained persistently positive (from 40 to 50 RU/mL), urine protein levels fluctuated at or above 2 g/day. Subsequently, the treatment strategy was modified to oral tacrolimus at a dose of 1.0 mg twice daily. The dosage was not adjusted during follow-up in other hospitals, but the blood drug concentration test results were no longer available. This adjustment led to a stabilization of plasma albumin levels at approximately 40 g/L and a reduction in urine protein to a nadir of 1 g/day. However, proteinuria subsequently rebounded, and anti-PLA2R antibody remained persistently positive (>30 RU/mL).

**Figure 1:**
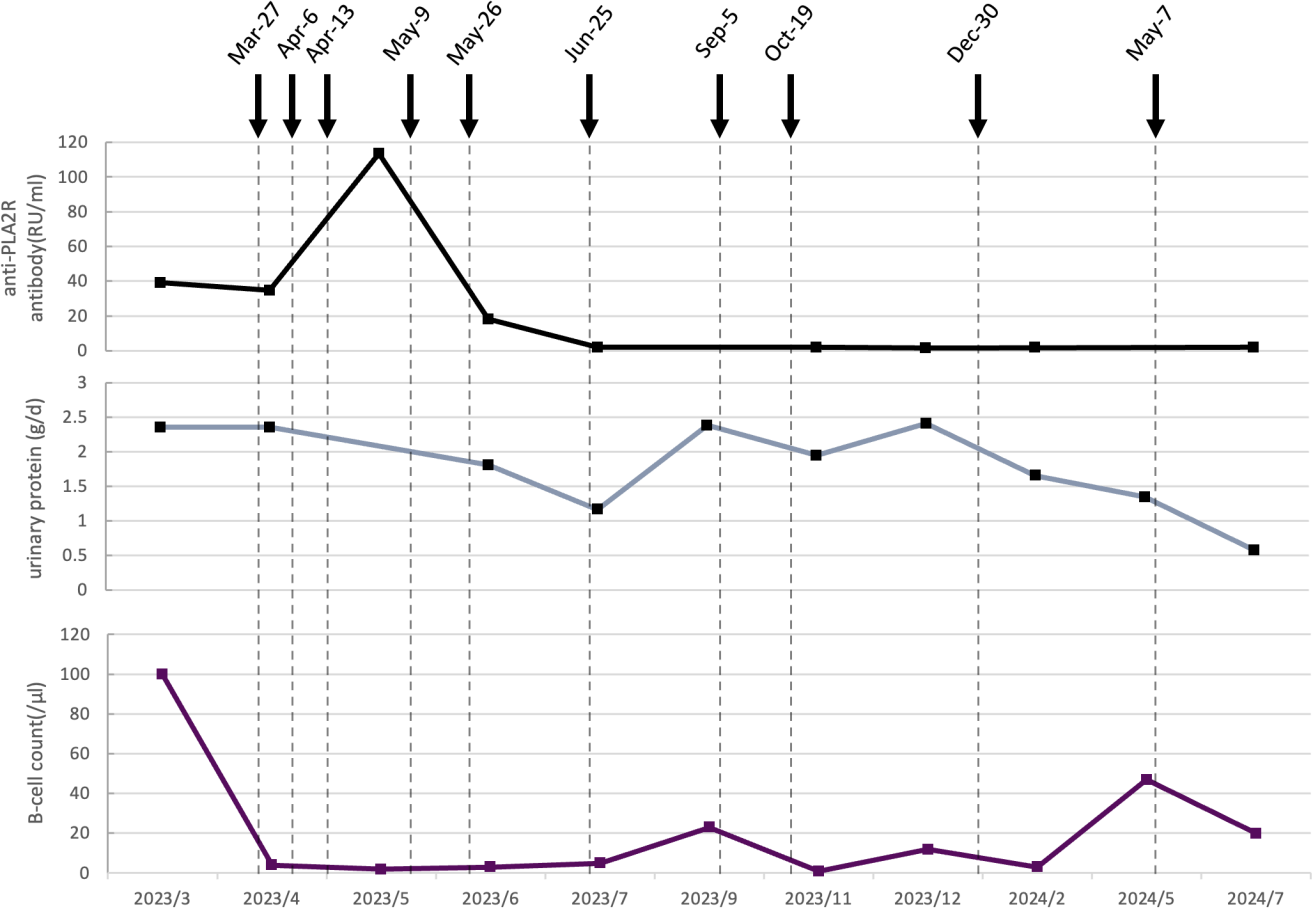
Dynamic changes of serological and clinical markers following ofatumumab treatment in Case 1. The black curve represents the anti-PLA2R antibody titer (RU/mL). The gray curve shows urinary protein (g/d). The purple curve displays the B cell count (/μL). Vertical dashed lines indicate specific clinical timepoints. Black arrows represent the time of ofatumumab administration, with each arrow corresponding to a single subcutaneous dose of 20 mg.

After 3 years since renal biopsy (March 2023), blood pressure was 115/70mmHg, laboratory findings showed plasma albumin of 39.4 g/L, urine protein of 2.2 g/day, anti-PLA2R antibody at 39.19 RU/mL, serum creatinine of 65.8 µmol/L and a total B-cell count of 100/μL. Although the patient’s renal function remained normal, he has sought treatment at multiple hospitals, yet anti-PLA2R antibody levels never reached a state of remission and urine protein exhibited recurrent elevations despite various treatment attempts. Ultimately, the patient sought care at our hospital and discontinued tacrolimus on his own initiative. Given the previous treatment history, CD20 monoclonal antibody therapy was recommended. According to the guidelines, rituximab is recommended as the first choice. However, he preferred an outpatient treatment approach and were unwilling to undergo hospitalization for medication administration, subcutaneous ofatumumab was selected as an alternative therapy. The treatment plan consists of an initial phase with 20 mg administered at weeks 0, 1, and 2, followed by a follow-up phase with 20 mg given at weeks 6, 8, and 12. Subsequent injections were administered depending on test results. The medication timeline is illustrated in **Figure 1**. No other treatment was received.

Following the sixth injection (July 2023), anti-PLA2R antibody converted to negative, urine protein decreased to 1.17 g/day, plasma albumin improved to 41.3 g/L, and the total B-cell count dropped to 5/μL. In light of these favorable responses, injections were temporarily halted. About 2 months later, anti-PLA2R antibody remained negative and plasma albumin was 40.5 g/L, but urine protein level rose to 2.39 g/day with a total B-cell count of 23/μL. Two additional doses of ofatumumab were administered at one-month intervals. 9 months after the first ofatumumab injection (December 2023), anti-PLA2R antibody remained negative and plasma albumin was 43.1 g/L, urine protein levels remained elevated at 2.41 g/day, with a total B-cell count of 12/μL, the ninth dose was given. Another 3 months passed (May 2024), the follow-up test revealed an increase in the total B-cell count to 47/μL with plasm albumin of 44.6 g/L and urine protein of 1.35 g/d, the tenth injection was administrated. 16 months after the first ofatumumab injection (July 2024), urine protein had further decreased to 0.58 g/day, the B-cell count was 20/μL, and anti-PLA2R antibody remained negative.

Although complete remission of urine protein was not achieved throughout the treatment, anti-PLA2R antibody remained persistently negative, renal function (within 66-95 µmol/L) and plasma albumin (within 38.4-46.5 g/L) levels remained normal, no drug-related adverse events were observed. Additionally, the treatment plan aligned with the patient’s preference for outpatient therapy, ensuring both clinical efficacy and convenience.

### Case 2

In October 2014, a 73-year-old man underwent a renal biopsy confirming stage 2 *P*MN. He had a history of hypertension, but no history of diabetes or kidney disease. A chest CT scan after admission showed chronic inflammatory changes. Initial laboratory investigations were urine protein 9.04 g/d, serum albumin 21.7 g/L, and normal serum creatinine(74.3 µmol/L). The patient presented with progressive generalized edema, which was the primary reason for seeking care at our institution. He was started on tacrolimus (1 mg twice daily), alongside nifedipine and metoprolol (Betaloc). Following two months of therapy, the patient exhibited a reduction in urine protein to 0.15 g/day and an increase in serum albumin to 41.6 g/L. Over the next 6 years, the patient remained complete remission and tacrolimus was gradually reduced to 0.5 mg every 3 days.

6 years after renal biopsy (August 2020), the patient experienced a relapse, with urine protein increasing to 6.71 g/day and serum albumin decreasing to 33.2 g/L. Blood pressure was 130/82mmHg. Tacrolimus was re-escalated to 1 mg twice daily, telmisartan (80mg daily) was added for anti-proteinuria therapy and blood pressure control. Although the blood concentration of tacrolimus (about 5–8 ng/ml) was within the therapeutic range, the treatment effect was not satisfactory as before. Urinary protein excretion ranged from a minimum of 0.88 g/day to a maximum of 5.38 g/day. Plasma albumin levels gradually increased and stabilized around 40 g/L. Serum creatinine remained relatively stable, ranging from 81 to 110 µmol/L. The anti-PLA2R antibody initially rose to 17.86 RU/mL but subsequently declined rapidly to a negative level.

**Figure 2:**
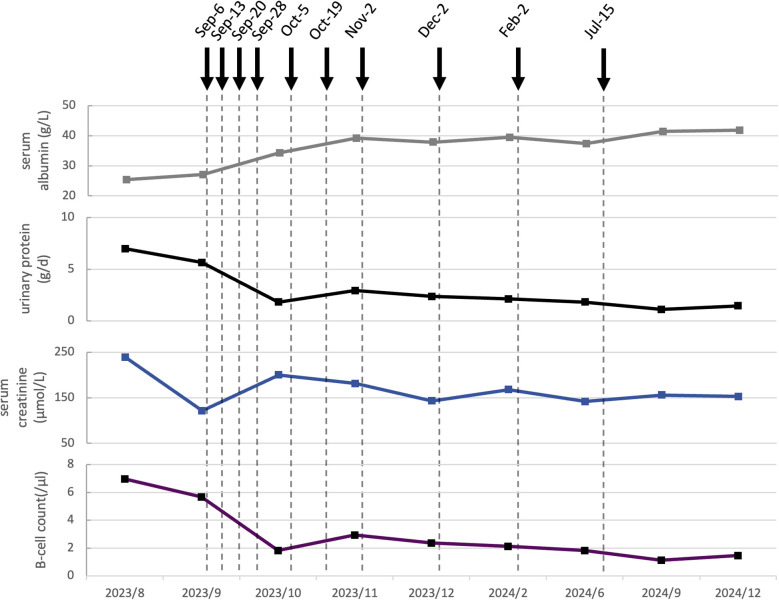
Dynamic changes of serological and clinical markers following ofatumumab treatment in Case 2. The gray curve indicates the serum albumin level (g/L). The black curve shows urinary protein (g/d). The blue curve represents serum creatinine (µmol/L). The purple curve displays B-cell count (/μL).Dashed vertical lines mark the dates of clinical visits. Solid black arrows denote the administration of ofatumumab, with each arrow indicating a single dose of 20 mg subcutaneously.

3 years later (August 2023), disease progression was evident with worsening urine protein (11.07 g/day), hypoalbuminemia (29 g/L), and deterioration of renal function (serum creatinine 157.3 µmol/L). Besides, over the past year, the patient developed generalized rashes that progressively worsened and were accompanied by scrotal swelling. He was subsequently diagnosed with severe atopic dermatitis. Although the anti-PLA2R antibody was 1.82 RU/mL, CD20 monoclonal antibody therapy was recommended due to the limited response to tacrolimus, as indicated by a marked increase in serum creatinine and urine protein, significant hypoproteinemia and edema. However, the patient declined this treatment option. Treatment was supplemented with oral prednisone (30 mg daily) and Chinese patent medicine aimed at improving renal function based on the tacrolimus treatment. Dermatological symptoms were managed with Dupilumab injections. One month later, urine protein quantification decreased to 6.96 g/day, yet cutaneous symptoms and edema persisted, plasma albumin continued to decline (25.4 g/L), and acute renal function deterioration was noted (serum creatinine: 239.2 µmol/L). We recommended treatment with a CD20 monoclonal antibody again, rituximab would normally be the first choice; however, it may cause allergic reactions during the initial infusion, most commonly manifesting as rash or urticaria. Considering the patient’s current severe rash and their reluctance to risk further aggravation, subcutaneous ofatumumab was initiated. Compared with Case 1, this patient presented with a more severe clinical profile, characterized by higher levels of urinary protein and more pronounced impairment of renal function. Thus, the treatment regimen consisted of 20 mg administered at weeks 0, 1, 2, 3, and 4, followed by additional doses every 2 to 4 weeks, or at longer intervals depending on comprehensive evaluation of test results and clinical symptoms. The medication timeline is illustrated in **Figure 2**. Besides, tacrolimus was discontinued because of worsening renal function and ineffective treatment.

Notably, both edema and scrotal eczema showed marked improvement following the second administration. The laboratory results indicated a urinary protein of 2.47 g/day, a plasma albumin of 29.7 g/L, a reduction in B-cell count from 82/μL to 17/μL, and a serum creatinine level of 125 μmol/L. Prednisone was tapered to 20 mg. By the fifth dose, urinary protein had decreased to 1.83 g/day, plasma albumin had increased to 34.3 g/L, serum creatinine levels exhibited fluctuations but remained lower than maximum value. Prednisone was tapered to 15 mg. After the eighth administration, the scrotal eczema had completely resolved. Laboratory evaluations revealed a plasma albumin level of 37.9 g/L, serum creatinine of 143 µmol/L, urinary protein of 2.37 g/day and B-cell count of 2//μL. A total of ten doses were administered. Follow-up post-treatment demonstrated persistently negative anti-PLA2R antibody, persistent proteinuria below 1.5 g/day; serum creatinine levels remaining under 160 µmol/L, and plasma albumin consistently above 40 g/L. Prednisone was tapered to 10 mg. No adverse events were observed throughout the course of ofatumumab therapy.

## Discussion

Both cases showed clinical improvement following ofatumumab treatment, including PLA2R antibody seroconversion to negative in Case 1, stabilization of renal function in Case 2, and reductions in proteinuria and B-cell counts in both patients. Given its comparable therapeutic efficacy and the convenience of subcutaneous administration, ofatumumab may become a valuable option for managing PMN, particularly in patients with limited treatment options.

One of the most significant advantages of subcutaneous ofatumumab is its convenience in administration, which can improve patient compliance and reduce the need for frequent hospital or clinic visits associated with intravenous therapies. As in Case 1, the patient was unwilling to be hospitalized for work reasons, and subcutaneous injection largely solved the problem of difficulty in medication. A systematic review of patients with various chronic immune-mediated conditions found that most patients prefer subcutaneous injections over intravenous infusions when therapeutic efficacy is comparable (15). Rituximab infusions typically last several hours and require specialized facilities and monitoring, while a subcutaneous injection of ofatumumab can be completed in minutes, with only brief observation needed after the first dose. From the patient’s perspective, this improvement in quality of life is substantial. On the other hand, the ability to administer the drug in an outpatient clinic or at home means fewer hospital visits, which has collateral benefits such as lower risk of hospital-acquired infections and reduced travel burden for patients (particularly relevant for those who live far from treatment centers or have mobility issues).

Another advantage of subcutaneous ofatumumab is its favorable safety profile. Infusion-related reactions (IRRs) are one of the main concerns of rituximab, especially during the first infusion (16). A systematic review reported that 16 of 33 cases with rituximab-related serum sickness had the classic triad of allergic reactions, namely fever, rash, and arthralgia (17). In recent years, obinutuzumab showed good efficacy in patients who have an inadequate response to rituximab, yet it also increased toxicity (18). Some experts recommend ofatumumab(intravenous injection) for patients who cannot receive rituximab due to adverse reactions (19). In patients with up to 3.5 years of exposure, subcutaneous ofatumumab was well tolerated with no cases of opportunistic infections or progressive multifocal leukoencephalopathy, and a low risk of malignancies (20). In an open-label extension study involving nearly 2,000 patients on continuous subcutaneous ofatumumab, the infection rate remained stable, with no increase in serious infections or malignancies over time (21). In a minority of RMS patients, subcutaneous ofatumumab only causes mild to moderate injection-site reactions (e.g., localized redness, itching, or swelling) (14). As we can see, no adverse events occurred in our cases. The lower peak serum concentration and slower absorption associated with subcutaneous administration may help mitigate acute cytokine release and reduce the incidence of IRRs commonly observed with intravenous anti-CD20 therapies, thereby improving overall tolerability. Moreover, in Case 2, the patient’s long-standing atopic dermatitis, including scrotal eczema, showed marked improvement following the initiation of subcutaneous ofatumumab. Although there is currently no published evidence directly supporting the use of ofatumumab in atopic dermatitis, this observation represents an unexpected and potentially meaningful finding, suggesting a possible ancillary benefit that warrants further investigation.

The efficacy of ofatumumab is our primary concern. About 23% to 43% of PMN patients treated with rituximab develop anti-rituximab antibodies (22). The growing number of rituximab-resistant PMN cases highlights the need for additional substitute treatment options (23). Small case series of rituximab-resistant or -intolerant PMN showed that a single intravenous dose of ofatumumab (50–300 mg) induced remission with reduced proteinuria and stable or improved glomerular filtration rate (GFR) (24). In another international retrospective multicenter study involving 34 PMN patients with anti-rituximab antibodies, ofatumumab(300 mg on day 1 and 1000 mg on day 8, except for one on day 21 additionally) were more likely to achieve clinical remission compared to those treated with rituximab (25). Ofatumumab is considered to have more effective complement-dependent cytotoxicity than other monoclonal antibodies (mAbs), as its antigen binding site is very close to the cell membrane and binds more tightly to the CD20 residue by interacting with epitopes rich in hydrophobic residues (26). This binding allows ofatumumab to securely anchor to CD20, even if part of the epitope is obscured; more importantly, it sterically hinders the approach of complement regulatory proteins to the cell surface once ofatumumab is bound (27). In contrast, rituximab binds a more distal epitope that involves only the large loop of CD20. Besides, ofatumumab’s fully human structure significantly reduces immunogenicity and the formation of anti-drug antibodies (14). This reduces the likelihood of reduced efficacy due to antibody formation, a crucial advantage in patients who have experienced rituximab hypersensitivity (28). Although neither of our patients had previously received rituximab, nor tested for anti-rituximab antibodies, these data provide a theoretical justification for selecting ofatumumab in similar future scenarios. Of course, further research is needed.

In previous case studies, ofatumumab was administered intravenously at varying doses ranging from 50 mg to 1000 mg including in *P*MN patients (24, 25, 29). However, in our cases, we used a subcutaneous dose (20mg) for the first time. This choice was motivated by (i) the patients’ strong preference to avoid prolonged infusions and the possible risk of IRRs with rituximab (16, 17); and (ii) the logistical advantage of a rapid, outpatient (or even home-based) subcutaneous injection. This context differs from classic “rituximab-resistant” PMN but reflects real-world scenarios in which an alternative anti-CD20 agent is needed before rituximab is even attempted. As for the dosage of drug, we refer to the use of ofatumumab in RMS as previously described (14). In fact, low-dose anti-CD20 monoclonal antibody therapy has been demonstrated to selectively deplete disease-relevant B-cell subsets and ameliorate autoimmune activity without significantly impairing immune surveillance (30). Several studies explored treatment strategies using low-dose anti-CD20 monoclonal antibodies to achieve an optimal balance between efficacy and safety. For instance, a low-dose rituximab regimen (100 mg intravenously per month) was administered to patients with anti-PLA2R-positive PMN, resulting in remission in 84% of cases (31). Earlier studies found that low-dose rituximab(100mg) induced long-term remission of Graves orbitopathy without further therapy (32). These studies provide a theoretical basis for our dosage selection, supporting an approach that optimizes therapeutic efficacy while minimizing costs and adverse effects. Our treatment results showed that case 1 demonstrated serological remission, with the persistently positive PLA2R antibody turning negative and proteinuria stabilizing at approximately 1g. Case 2 improved renal function with a reduction in quantitative proteinuria, an increase in plasma albumin, a continued negativity of anti-PLA2R antibodies, and—most notably—a marked improvement in the rash that had persisted for over a year, which has not been reported before. These results support the feasibility of low-dose subcutaneous ofatumumab; nonetheless, long-term surveillance and larger prospective cohorts are required to confirm durability, safety, and the optimal dosing schedule.

Our report presents a novel exploration of low-dose subcutaneous ofatumumab in the treatment of PMN, with strengths in safety, patient convenience and immunological efficacy. The evidence so far indicates that ofatumumab is an effective therapy especially for patients in whom glucocorticoids and immunosuppressants therapy is ineffective. However, there were some limitations in our study. First, B-cell depletion appeared to be suboptimal, and complete remission of proteinuria has not yet been achieved based on the current observations. Second, the small number of cases limits the generalizability of our findings to the broader patient population. It is also important to consider the appropriate timing for administering additional doses of the drug. Furthermore, there were shortcomings in our treatment protocol, including the failure to initiate first-line antiproteinuric therapy-such as sodium-glucose cotransporter-2 (SGLT2) inhibitors and mineralocorticoid-receptor antagonists-during the early phase, as recommended by current clinical guidelines. Nevertheless, these two cases may provide some inspiration for future treatments, such as more diverse choices for CD20 monoclonal antibodies. In future studies, larger patient cohorts and longer follow-up durations will be necessary to optimize dosing strategies and administration schedules, as well as to further validate the efficacy of the treatment.

## Data Availability

The original contributions presented in the study are included in the article/supplementary material. Further inquiries can be directed to the corresponding author.
